# Congenital Radioulnar Synostosis Review: Recommendations and Treatment Outcomes

**DOI:** 10.3390/children11111317

**Published:** 2024-10-30

**Authors:** Sergi Alabau-Rodriguez, Jose Felix Garrido Ferrer, Xavier Bulló Mir, Lidia Ana Martín Dominguez, Albert Pardo Pol, Francisco Soldado Carrera

**Affiliations:** 1 Institut Català de Traumatologia i Medicina de l’Esport (ICATME), Hospital Universitari Quiròn-Dexeus, Universitat Autònoma de Barcelona, 08028 Barcelona, Spain; info@icatme.es (X.B.M.); enfermeriamanocodo@icatme.com (L.A.M.D.); albert.pardo93@gmail.com (A.P.P.); 2 Consorcio Hospital General Universitario, 46014 Valencia, Spain; garrido.jos1@gmail.com; 3 Royal Academy of Medicine of Catalonia, Carrer del Carme, 47, Ciutat Vella, 08001 Barcelona, Spain; 4 Unidad de Plexo Braquial y Microcirugía, Hospital de Nens, 08009 Barcelona, Spain; atpaciente.hmn@hmhospitales.com

**Keywords:** radioulnar synostosis, radius, ulna, congenital

## Abstract

**Background/Objectives:** Congenital radioulnar synostosis (CRS) is a rare congenital disorder of the elbow joint caused by the abnormal fusion of the radius and ulna during fetal development, leading to limited forearm rotation and functional impairment. This narrative review aims to summarize the key aspects of diagnostic suspicion, treatment options, and lifestyle management strategies for individuals affected by CRS. **Relevant sections**: While CRS often occurs sporadically, there are familial cases with an autosomal dominant inheritance pattern. The diagnosis is established through a combination of clinical evaluation and radiological imaging, which confirms the presence and extent of the synostosis. Identifying the specific type and severity of CRS is critical for management decisions. Surgical interventions are considered based on factors such as the patient’s age, level of functional limitation, and symptom severity, while conservative treatment may be appropriate for cases with mild impairment. **Discussion:** Various surgical techniques have been described, but derotation osteotomy has emerged as a preferred option due to its predictable improvement in forearm function. Nevertheless, surgical treatment poses challenges, including potential complications like nerve injury and recurrence of deformity. Cultural and individual considerations, such as the desired forearm position, must be addressed to achieve optimal outcomes aligned with the patient’s lifestyle and needs. **Conclusions:** Managing CRS requires a nuanced and individualized approach, recognizing the unique challenges each patient presents. This review highlights the importance of continuous research to refine diagnostic and therapeutic strategies, ultimately aiming to enhance functional outcomes and quality of life for CRS patients.

## 1. Introduction

Congenital radioulnar synostosis (CRUS) is a rare anomaly that affects the normal development of the forearm. It is characterized by the abnormal fusion of the radius and ulna, leading to limitations in daily activities due to substantial implications for elbow joint function and mobility [1,2,3].

As we advance in our understanding of CRUS, our aim is not only to enhance current clinical management but also to promote ongoing research to refine therapeutic approaches and optimize long-term outcomes.

After a review of the articles published in the literature, the objective is to present clinical, radiological, and functional aspects, as well as various treatment modalities, providing a comprehensive approach to current knowledge and offering healthcare professionals an informative and updated guide on this uncommon yet relevant orthopedic condition.

## 2. Purpose

The aim of this study is to describe a rare congenital pediatric pathology, intending to raise diagnostic suspicion among professionals. Likewise, to delve into the characteristic clinical features and physical examination and emphasize the importance of a diagnosis supported by complementary tests to enhance the quality of life for patients.

Furthermore, to present the broad spectrum of surgical interventions, more closely related to pediatric orthopedic specialists. The controversy regarding how and when to perform surgery, as well as the improvement and the subsequent likelihood of complications, remains a field of study where exploration is needed to establish an optimal treatment algorithm.

Lastly, to encourage professionals to conduct early diagnostic screening during school ages through a more detailed joint physical examination since, as discussed below, persistent functional limitations after a late diagnosis are relevant.

Scientific article databases such as PubMed, Cochrane, and UpToDate have been utilized. The keywords used were: radioulnar synostosis; radius; ulna; and congenital. Once the articles were obtained through this search strategy, we selected the most relevant ones for each section of the diagnostic and therapeutic process, including both classic articles on the management of this condition as well as the most recent ones, in order to provide a comprehensive and generalized view.

## 3. Relevant Sections

Congenital radioulnar synostosis (CRUS) is a congenital elbow malformation involving abnormal prenatal segmentation during development, resulting in fibrous or bony bridging that restricts forearm rotation [1,2].

### 3.1. Etiology

CRUS has a sporadic and multifactorial etiology influenced by genetic factors during longitudinal segmentation. Although the exact cause and inheritance pattern of CRUS are unknown, some studies have reported autosomal dominant inheritance with common incomplete penetrance [4,5,6]. During embryological development, the upper limb bud arises from the non-segmented body wall between days 25 and 28, with the elbow developing around day 34 and the humerus and ulna developing around day 37. The cartilaginous analogs of the humerus, radius, and ulna are connected before segmentation. Therefore, for a brief period, the radius and ulna share a common perichondrium [7]. CRUS results from the failure of longitudinal separation and the persistence of cartilaginous anchoring of the forearm’s perichondrium during the seventh week of gestation [8,9,10]. This bridge commonly ossifies into a bony synostosis but can also remain unossified as a fibrous synostosis depending on the duration and severity of the insult [11].

There is a developmental relationship between posterior dislocation of the radial head and proximal radioulnar fusion. Both abnormalities can occur in the same patient and have been considered different clinical manifestations of the same primary radioulnar differentiation/segmentation anomaly [12,13]. Moreover, both abnormalities can also be observed in different patients with the same genetic diagnosis, supporting the idea that these defects are developmentally related [13]. This contrasts with patients with transverse forearm defects who may also show dislocation of the radial head but in an anterior or lateral direction. This dislocation direction is observed in disorders such as multiple osteochondromatosis and various mesomelic dysplasias or as a result of trauma [13].

### 3.2. Epidemiology and Associated Syndromes

The genetic pattern and inheritance of the condition are variable. There are clear cases of autosomal dominant inheritance, as well as de novo mutations or associations with other genetic diseases featuring mutations in which synostosis occurs.

Although CRUS is a rare congenital disease, it is the most common congenital disorder affecting the elbow and forearm joint [3], being bilateral in 60–80% of cases [4,5,6].

It is usually an isolated condition without associated injuries [2,3,14,15]. However, one-third of cases show some other abnormality, such as congenital syndromes (arthrogryposis, Apert syndrome, Carpenter syndrome, Williams syndrome, and Antley–Bixler syndrome), chromosomal abnormalities (Klinefelter syndrome and other mosaicism of tetrasomy or pentasomy), upper limb abnormalities (brachydactyly, polydactyly, syndactyly, thumb aplasia, and Madelung deformity), anomalies of the lower limbs (hip dislocation and tarsal bone synostosis) hematological abnormalities observed in patients with germline variants of HOXA11 or MECOM (from thrombocytopenia to myelodysplastic syndrome and global bone marrow failure), or other conditions such as hip dislocation, clubfoot, osteogenesis imperfecta, and cardiac abnormalities urinary tract abnormalities [2,16,17]. The most recent and well-known genetic mutation associated with congenital radioulnar synostosis is SMAD6, specifically the interstitial microdeletion 8q22.2q22.3., characterized by moderate to severe intellectual disability, seizures, distinct facial features, and skeletal abnormalities [16].

### 3.3. Clinical and Physical Examination

In CRUS patients, the forearm is typically in a pronated position, and they have difficulty performing activities that require supination. The condition generally does not cause pain [3,4,14,18]. In a smaller number of cases, forearm pronation and supination can cause a manifestation known as “snapping elbow”, especially in those cases with a less mature synostosis membrane [14,19].

Functional disabilities due to the lack of forearm rotation vary depending on the forearm’s position. When the forearm is positioned nearly in a neutral position, joint laxity in the hand, wrist, and shoulder allows for the performance of most daily living activities. These compensatory mechanisms can delay diagnosis until primary school age [20].

However, some patients have a hyperpronated forearm that can cause difficulties in some daily activities, such as eating with chopsticks, catching balls, washing the face and hands, dressing, grasping objects in the palm, using soap, and opening doors [21], with the latter being less tolerated.

In cases where CRUS manifests bilaterally with significant hyperpronation (>60–70°), it can result in severe disability. In such cases, the total loss of pronosupination leads to serious functional limitation not only in sports but in daily life, as adaptations are significantly poorer [15].

### 3.4. Radiological Study

For complementary studies, conventional two-projection X-rays of the forearm are usually sufficient for most patients.

Some patients will show complete synostosis, while others will have a “partially separated” radial head. The latter likely represents a later developmental insult (failure of separation). Therefore, radiological examination reveals fusion or synostosis, with four possible scenarios [15] based on the degree of ossification and the appearance of synostosis in X-rays, the length of the synostosis, and the position of the radial head (Table 1). However, no relationship was observed between any of these patterns and functions [15].

### 3.5. Treatment

The ideal treatment for congenital radioulnar synostosis aims to restore rotational function and prevents the recurrence of the bony bridge. Both conservative and surgical treatments are possible, but results are still controversial. Due to the low number of cases, conducting randomized controlled trials is challenging, and typically only case reports or case series are published [3].

Physiotherapeutic treatment is not indicated as it does not improve range of motion values [22]. On the other hand, the primary indication for surgical treatment is the limitation of daily activities, as there is no consensus on the forearm position beyond which surgery should be recommended.

This very fact is the reason for advocating conservative treatment. Therefore, for patients who do not experience daily functional limitations, do not present symptoms associated with the deformity, and for whom congenital radioulnar synostosis has no psychological or social impact, conservative treatment is the best alternative, with regular follow-up to monitor its progression [22].

#### 3.5.1. Surgery Indications

**Figure 1:**
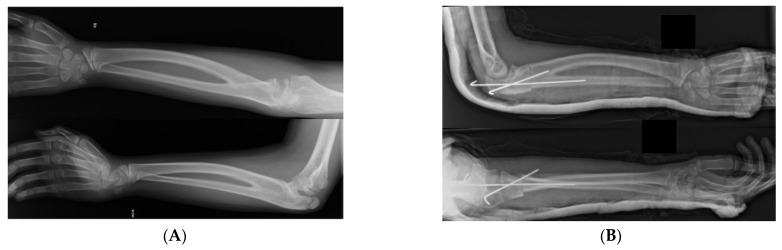
(**A**) Bilateral congenital radioulnar synostosis. Preoperative X-ray. (**B**) Osteotomy through the synostosis going simple and easy to fix with 2 KW. Postoperative X-ray.

Surgery is a common treatment for symptomatic patients with CRUS (Figure 1), but it is not required for most patients unless they have limitations of daily life activities. Mild deformity, minimal functional deficit (<60° of pronation), and adaptations developed for activities are contraindications for surgical procedures [3]. However, surgical treatment would be desirable in bilateral congenital radioulnar synostosis (CRUS) or in patients with a forearm fixed in more than 60° of pronation. The optimal age for surgical treatment is before school age, when the robust periosteum can support the cut radius and facilitate callus formation; thus, nerves and blood vessels can tolerate torsional deformity, avoiding vascular and postoperative complications [3,23].

Although many surgical methods have been reported aiming restoration of radius rotation around the ulna (synostosis resection and artificial biological tissue interposition), all these treatments have failed. Thus, the only effective and indicated surgery is the derotational osteotomy, when the forearm is placed in >60° of pronation or placed in supination [24].

#### 3.5.2. Surgical Principles

Historically, surgical separation of the synostosis and vascularized and non-vascularized interposition techniques to fill the interosseous space and prevent scar formation and resynostosis had theoretically been considered the ideal treatment. Although separation of the two bones can be achieved by mobilization surgery, gain in active rotation is usually slight in congenital cases and frequently results in recurrence of the ankylosis with unsatisfactory results.

Furthermore, inherent musculoskeletal disorders of congenital synostosis, such as bowing of the radius, hypoplastic radial head, aberrantly oriented local fibrous tissue, constricted interosseous membrane, and absence of powerful forearm supinator muscles, may also inhibit active forearm rotation.

Currently, the most accepted surgical technique in CRUS is a corrective rotational osteotomy because of its reliability and predictable results. The goal is to regain a neutral position of the forearm, allowing the performance of most activities through compensatory movements of the shoulder and hand [25], although pronosupination is not restored by not eliminating the synostosis region [21,26,27,28].

There are various types of osteotomies and fixation methods for CRUS. The standard is the osteotomy through the synostosis, going simple and easy to fix with 2 KW. The main complication might be compartmenting syndrome because of the torsion of the forearm soft tissues; thus, the patient should stay overnight. In older patients, as they have more rigid tissues, a segment of the synostosis might be respected in order to decrease forearm pressure after torsion. Fascia release might be indicated also. Osteotomy of both bones (radius and ulna) at different levels has favorable outcomes but necessitates two surgical scars and fixation with nails (with the associated risk of penetrating the growth plate) [29]. The advantages of a single osteotomy (either radius or ulna) over a double one include surgery simplicity with minimal complications and surgical scars [27]. A highly feared complication during the rotation process, aside from compartment syndrome, is the injury to the posterior interosseous nerve, as described by Hung et al. [28].

Despite this, numerous surgical options have been reported by various authors and summarized in Table 2.

Regarding the optimal forearm position after rotation, it involves controversial issues related to customs, dominance, the affected side and individual needs. For example, people in Western countries use knives and forks for eating and do not need complete forearm supination to do so. However, people in East Asia require forearm supination to use chopsticks and bring food to their mouths.

Currently, the widely accepted final forearm position is neutral to slightly pronated to treat patients with severe pronation (>60°) [26,28,31,33]. However, some authors prefer correcting the forearm position to 30° of supination in East Asian patients [34].

Green et al. suggested that the ideal position is 10° to 20° of supination in unilateral cases. Nevertheless, they found that if one forearm was placed in supination, it complemented the other in 30° to 45° of pronation, and the patient could perform tasks requiring both supination and pronation. Therefore, in bilateral cases, the best positions are 30° to 45° of pronation in the dominant forearm and 20° to 35° of supination in the nondominant forearm [35]. Other authors have reinforced this position, albeit with a lower degree of rotation, from 0 to 20 degrees of supination in the nondominant forearm and from 0 to 20 degrees of pronation in the dominant forearm [36,37]. Furthermore, a 30-degree supination of the dominant arm with the contralateral arm in a neutral position has also been advocated [38]. Therefore, there is controversy and variability regarding the final position, with the best approach being one that is individualized for each patient [39,40].

## 4. Conclusions

Congenital radioulnar synostosis represents a relatively uncommon pathology, frequently diagnosed belatedly due to an initial low suspicion index. A thorough examination of each patient is imperative, encompassing the analysis of associated syndromes alongside the recommendation for appropriate genetic assessment. The patient may follow a conservative treatment approach if their functional independence allows them to perform basic activities of daily living. Conversely, following diagnosis, surgical intervention becomes imperative in cases where patients experience limitations in essential daily activities. The determination of the ultimate limb position, achieved through osteotomy and derotation, necessitates consideration of functional exigencies as well as socio-cultural determinants.

## 5. Future Directions

Following this study and in line with the aforementioned objectives, the aim is to increase knowledge about a pathology studied over the years that still lacks diagnostic and treatment protocol. Functional limitations at such early ages may be tolerated due to the plasticity of patients in generating compensatory mechanisms. However, despite not being aware of the long-term results of new surgical treatments, finding a solution for them seems logical to improve their quality of life during adulthood.

Individualizing each patient, making an early diagnosis through screening in school check-ups, and finally, choosing the therapeutic approach as well as the target final position of the upper limbs are the four pillars supporting this study. Nevertheless, we consider a greater number of articles related to this pathology necessary to generate a substantial evidence algorithm.

A more sophisticated line of research would be to emphasize genetic studies, both prenatal and neonatal, to achieve prevention and prognosis for each patient’s condition. This approach would enable a multidisciplinary intervention from an early age.

## Figures and Tables

**Table 1:** Radiocubital synostosis types and description. Cleary and Omer’s classification.

Radiocubital Synostosis Type	Radiological Items
I	Fibrous Synostosis: Does not involve bone and is associated with a normal and articulated radial head.
II	Osseous Synostosis: Associated with a normal and articulated radial head.
III	Osseous Synostosis: With a hypoplastic radial head displaced backward.
IV	Short Osseous Synostosis: With a mushroom-shaped radial head displaced forward.

**Table 2:** Types of surgical treatment for congenital radioulnar synostosis.

Author	Year	Treatment
Sakamoto et al. [30]	2014	Interposition synostosis via vascularizes adipofascial tissue.
Horii et al. [27]	2014	A single osteotomy was performed at the radial diaphysis.
Hung et al. [28]	2008	Simultaneous osteotomy of the radius and ulna at different levels.
E.A. Wael et al. [26]	2007	Separate osteotomies of the radius and ulna at different levels.
Dalton et al. [31]	2006	Osteoclasis: Breaking the bone in the synostosis region.
Funakoshi et al. [32]	2004	Interposition synostosis via vascularizes flap tissue.
M. Ramachandran et al. [33]	2004	Interposition synostosis via vascularizes adipofascial tissue.

## Data Availability

The data presented in this study are available on request from the corresponding author. The data are not publicly available due to ethical standards.
